# Supplementary material for: Genetic Diversity of Cytochrome P450s CYP6M2 and CYP6P4 Associated with Pyrethroid Resistance in the Major Malaria Vectors Anopheles coluzzii and Anopheles gambiae from Yaoundé, Cameroon
Source: Genes (Basel). 2022 Dec 23;14(1):52. doi: 10.3390/genes14010052 (PMC9858699; doi:10.3390/genes14010052)
Supplement: Supplementary file 1 [file genes-14-00052-s001.zip › Supp figures captions.pdf]

Figure S1: Nucleotide sequence of the 881bp of upstream region of CYP6M2 showing (a) the regulatory sequences identify by GPMiner and (b) the transcription factors binding sites using Alggen.

Figure S2: PCR amplification of the upstream and full-gene region of *CYP6M2*.

Figure S3: Polymorphic sites and haplotypes of the *CYP6M2* upstream region in (a) *An. coluzzii* and (b) *An. gambiae* hybrid from F4.

Figure S4: Polymorphic sites and haplotypes of the full –gene length of *CYP6M2* in (a) *An. coluzzii* and (b) *An. gambiae* hybrid from F4.

Figure S5: Sequencing of the portion of the full *CYP6M2*-gene length spanning the A392S mutation. *(a) Sequence alignment of the full CYP6M2-gene length at the A392S point mutation in HR, HS and Kisumu susceptible laboratory strain; (b) amino-acid change of the full CYP6M2-gene length at the A392S point mutation according to their phenotype and (c) Chromatogram traces showing the two genotypes at the 392-codon position.*

Figure S6: Nucleotide sequence of the 868 bp of the upstream region of *CYP6P4* showing (a) the regulatory sequences identify by GPMiner and (b) the transcription factors binding sites using Alggen.

Figure S7: PCR amplification of the putative promoter and full-gene region of *CYP6P4.*

Figure S8: Polymorphic sites and haplotypes of the *CYP6P4* upstream region in (a) *An. coluzzii* and (b) *An. gambiae* hybrid from F4.

Figure S9: Polymorphic sites and haplotypes of the 1,051bp fragment of *CYP6P4* gene in (a) *An. coluzzii* and (b) *An. gambiae* hybrid from F4.

Figure S10: **Sequencing of the portion of the full *CYP6P4*-gene length spanning the all the mutation found.** *(a) Sequence alignment of the full CYP6P4-gene length at point mutation in HR, HS and Kisumu strain; (b) amino-acid change at the C168S point mutation according to their phenotype.*

Figure S11: Representative diagram of DNA-based assay to genotype a key mutation in *An. gambiae CYP6M2*. *(a) CYP6M2 upstream region: alignment of sequences showing differences by resistance phenotype including the deletion of 7bp found in HR and HS groups link to the G/A variant generating a restriction site for the BsrDI restriction enzyme; a schematic representation of the CYP6M2pr PCR-RFLP illustration digestion of the PCR amplicon and genotyping results for F4 field-resistant from Nkolondom and Susceptible Kisumu crossing. (b) CYP6M2 gene region: alignment of sequences showing A392S-mutation differences by resistance phenotype; a schematic representation of the CYP6M2g AS-PCR illustration digestion of the PCR amplicon and genotyping results for F4 field-resistant from Nkolondom and Susceptible Kisumu crossing.*

Figure S12: Representative diagram of DNA-based assay to genotype a key mutation in *An. gambiae CYP6P4*. *(a) CYP6P4 upstream region: alignment of sequences showing differences by resistance phenotype linked to the A/T (A-273-T) variant generating a restriction site for the PvuII restriction enzyme; a schematic representation of the CYP6P4pr PCR-RFLP illustration digestion of the PCR amplicon and genotyping results for F4 field-resistant from Nkolondom and Susceptible Kisumu*

crossing. CYP6P4 gene region: (b) alignment of sequences showing the differences in codon 144 (C-432-T) by resistance phenotype; a schematic representation of the CYP6P4g RFLP-PCR illustration digestion of the PCR amplicon and genotyping results for F4 field-resistant from Nkolondom and Susceptible Kisumu crossing; (c) alignment of sequences showing C168S-mutation differences by resistance phenotype; a schematic representation of the CYP6P4g AS-PCR illustration digestion of the PCR amplicon and genotyping results for F4 field-resistant from Nkolondom and Susceptible Kisumu crossing.
