# Supplementary material for: Genetic Diversity of Cytochrome P450s CYP6M2 and CYP6P4 Associated with Pyrethroid Resistance in the Major Malaria Vectors Anopheles coluzzii and Anopheles gambiae from Yaoundé, Cameroon
Source: Genes (Basel). 2022 Dec 23;14(1):52. doi: 10.3390/genes14010052 (PMC9858699; doi:10.3390/genes14010052)
Supplement: Supplementary file 1 [file genes-14-00052-s001.zip › Supp tables.pdf]

Table S1: Primers of the promoter region of *CYP6M2* and *CYP6P4*

Primer name	Sequence 5’-3’	Product size	Annealing T°C
6M2p3i-Fw	5’-AGTACTTCACAGCGCCCAAC-3’	1 020bp	58°C
6M2pr3-Rv	5’-CACCACGATCCTGCCAGTAG-3’		
6M2full-Fw	5’-GTCGGTGGACAGTCAAATTCA-3’	1 600bp	56°C
6M2full-Rv	5’-CTAAATCTTATCCACCTTCAA-3’		
CYP6P4.pr1-Fw	5’-GACACCGTACACGTTTCTGC-3’	835bp	65°C
CYP6P4.pr1-Rv	5’-ATGTACGATGCGTGCTTGGA-3		
CYP6P4.ful1-Fw	5’-GCGCATGAAAGCTAGGGAT-3’	1 600bp	67°C
CYP6P4.ful2-Rv	5’-ACAGTACACTTACAATTCCTTCGG-3’		

***Fw**, primers forward, **Rv**, primers reverse.*

Table S2: Specific primers of *CYP6M2* and *CYP6P4* diagnostic assays

Mutation	PCR type	Primers/enzymes name and sequence	Annealing T°C	Products size
A/G in *CYP6M2* promoter	RFLP-PCR	6M2pfw: GAAGCCGATGCTTGAGATAAGA<br>6M2prv: CGTGTGTGTCAGGATGAGTT<br>BsrDI: GCAATGNN (incubation, 65°C)	58	D/D: Deletion carriers at 545bp (undigested)<br>I/I: Insertion carriers 2 band 382bp and 167bp
A392S-CYP6M2	AS-PCR	6M2fw: TGATGAGCAAAGACGGAGAGAAGT<br>6M2rv: CATACCGAATCGCAGCCCAAC<br>A392fw: CGGTACGAAATCCGTCCTGGtGG<br>392Srv: GGATCATGACGGCTGTACCcGA	50	392S/S at 527bp<br>A/A392 at 202bp<br>A392S-RS, two bands
A/T in *CYP6P4* promoter	RFLP-PCR	6p4p1fw: TCGATCCGAAATCGTTCATACTC<br>6p4p1rv: CCGCGTTTACTTGATGGTGTGAG<br>PvuII: CAGCTG (incubation, 37°C)	58	D+/D+ (T/T): Deletion carriers at 548bp (undigested)<br>D-/D-: Insertion carriers 2 band 374bp and 168bp
c/t in in CYP6P4-gene at codon 144	RFLP-PCR	6p4-ful1fw: GCGCATGAAAGCTAGGGAT<br>6p4-ful1rv: GCCAGCTCGTTCATCGTCAG<br>EagI: CGGTCG (incubation, 37°C)	61,5	t/t at 600bp (undigested)<br>c/c, two bands at 357bp and 269bp
C168S-CYP6P4	AS-PCR	6p4-ful1fw: GCGCATGAAAGCTAGGGAT<br>6p4-ful1rv: GCCAGCTCGTTCATCGTCAG<br>502Arv: CCTTCATCTCAATCTCGCGGTGcCT<br>502Tfw: CTTGAAGTATATGAACGAGCtCT	57	A168A at 614bp<br>T168T at 426bp<br>A168T-RS, two bands

fw, forward primer; rv, reverse primer; D, Deletion; I, insertion; S, Serine; A, Alanine; C, Cysteine; S, Serine; t, thymine; a, adenine; c, cytosine;

Table S3: Genotype and allele frequencies of *1014F kdr* mutations in *An. gambiae* and *An. coluzzii* populations

Population	Genotype			N	2N	Allele	
	FF	FL	LL			f(F)	f(L)
An. gambiae							
F_0	30	0	0	30	60	1.00	0.00
HR F_4	40	18	0	58	116	0.84	0.16
HS F_4	1	15	37	53	106	0.16	0.84
An. coluzzii							
F_0	26	4	0	30	60	0.93	0.07
HR F_4	18	12	0	30	60	0.80	0.20
HS F_4	0	4	26	30	60	0.07	0.93

***FF**, homozygous resistant, **FL**, heterozygous resistant, **LL**, homozygous susceptible.*

Table S4: Frequency of key mutations found and genetic variability parameters of *CYP6M2* per allele in *An. gambiae*

Pop.	Region	Position	Sample	Ref. seq.	Mut. Seq.	Aa change	Type of mut.	Mutation freq. (%)	n/N
An. gambiae	upstream	Indel[#]	Kisumu	T(ins.)	C(del.)	n.a	n.a	0.00	0/3
			HS (F4)	T(ins.)	C(del.)	n.a	n.a	0.83	1/6
			HR (F4)	T(ins.)	C(del.)	n.a	n.a	0.88	1/8
	Full-gene	1 252 (codon 392)	Kisumu	**G**CC	**T**CC	A/S	NSyn	0.00	0/7
			HS (F_4)	**G**CC	**T**CC	A/S	NSyn	0.57	4/7
			HR (F_4)	**G**CC	**T**CC	A/S	NSyn	0.71	5/7
CYP6M2		**Allele**	**2n**	**S**	**H**	**Hd**	**π**	**D**	**D***
		392S	18	25	11	0.889	0.0059	1.028	1.572
		392A	24	33	9	0.899	0.0089	2.074*	1.668**
		All	42	51	20	0.948	0.0124	2.124*	1.929**

2n, number of sequences (2n = Sequences were unphased because of the observed heterozygosity); D, Tajima's statistics; D *, Fu and Li's statistics; H, number of haplotypes; Hd, haplotype diversity; Syn, Synonymous mutations; Nsyn, Non-synonymous mutations; π, nucleotide diversity; S, number of polymorphic sites; pKa: Synonymous polymorphism per site; pKs: non-Synonymous polymorphism per site; * = P < 0.05; ** = P < 0.02; n=number of samples containing mutant allele; N = total number of successfully sequenced samples; n.a, non-available; [#], associated to a point mutation at 322 position.*

Table S5: Frequency of key mutations found and genetic variability parameters of the intergenic region between *CYP6P4* and *CYP6P5* per allele in *An. gambiae*

Pop.	Region	Position	Sample	Ref. seq.	Mut. Seq.	Mutation freq. (%)	n/N
An. gambiae	promoter	Indel#	Kisumu	**A**	**T**	0.14	1.5/8
			HS (F_4)	**A**	**T**	0.44	4/9
			HR (F_4)	**A**	**T**	1.00	6/6
CYP6P4pr	**Allele**	**2n**	**S**	**H(Hd)**	**π**	**D**	**D***
	273-T	21	5	5(0.595)	0.0013	-0.618	1.339
	273-A	23	19	7(0.834)	0.0094	2.108*	1.374**
	All	44	19	11(0.838)	0.0083	2.047*	1.655**

pr, promoter; 2n, 2n, number of sequences (2n = Sequences were unphased because of the observed heterozygosity); S, number of polymorphic sites; H, number of haplotypes; Hd, haplotype diversity; π, nucleotide diversity; D, Tajima's statistics; D *, Fu and Li's statistics; * = P < 0.05; ** = P < 0.02; n=number of samples containing mutant allele; N = total number of successfully sequenced samples; #, associated to a point mutation at 273 position.*

Table S6: Frequency of key mutations found and genetic variability parameters of the upstream region of mutation per allele in *An. gambiae*

Pop.	Region	Position	Sample	Ref. seq.	Mut. Seq.	Aa change	Type of mut.	Mutation freq. (%)	n/N
An. gambiae	Full-gene	432 (codon 144)	Kisumu	GG**C**	GG**T**	G/G	Syn	0.20	2/10
			HS (F_4)	GG**C**	GG**T**	G/G	Syn	0.28	4.5/9
			HR (F_4)	GG**C**	GG**T**	G/G	Syn	0.95	9.5/10
		502 (codon 168)	Kisumu	**T**GC	**A**GC	C/S	NSyn	0.15	1.5/10
			HS (F_4)	**T**GC	**A**GC	C/S	NSyn	0.27	2.5/9
			HR (F_4)	**T**GC	**A**GC	C/S	NSyn	0.70	7/10
CYP6P4		**Alleles**	**2n**	**S**	**H**	**Hd**	**π**	**D**	**D***
		144-T	32	17	19	0.920	0.005	0.665	1.365
		144-C	26	13	15	0.950	0.005	1.854	1.330
		168S	22	12	11	0.861	0.0033	1.096	1,470
		168C	36	14	22	0.968	0.0053	2.128*	1,441**
		All	58	17	33	0.970	0.0062	2.385*	1.632**

2n, number of sequences (2n = Sequences were unphased because of the observed heterozygosity); D, Tajima's statistics; D *, Fu and Li's statistics; H, number of haplotypes; Hd, haplotype diversity; Syn, Synonymous mutations; Nsyn, Non-synonymous mutations; π, nucleotide diversity; S, number of polymorphic sites; pKa: Synonymous polymorphism per site; pKs: non-Synonymous polymorphism per site; * = P < 0.05; ** = P < 0.02; n=number of samples containing mutant allele; N = total number of successfully sequenced samples.*
