# Supplementary figures and images for: Genetic Diversity of Cytochrome P450s CYP6M2 and CYP6P4 Associated with Pyrethroid Resistance in the Major Malaria Vectors Anopheles coluzzii and Anopheles gambiae from Yaoundé, Cameroon
Source: Genes (Basel). 2022 Dec 23;14(1):52. doi: 10.3390/genes14010052 (PMC9858699; doi:10.3390/genes14010052)

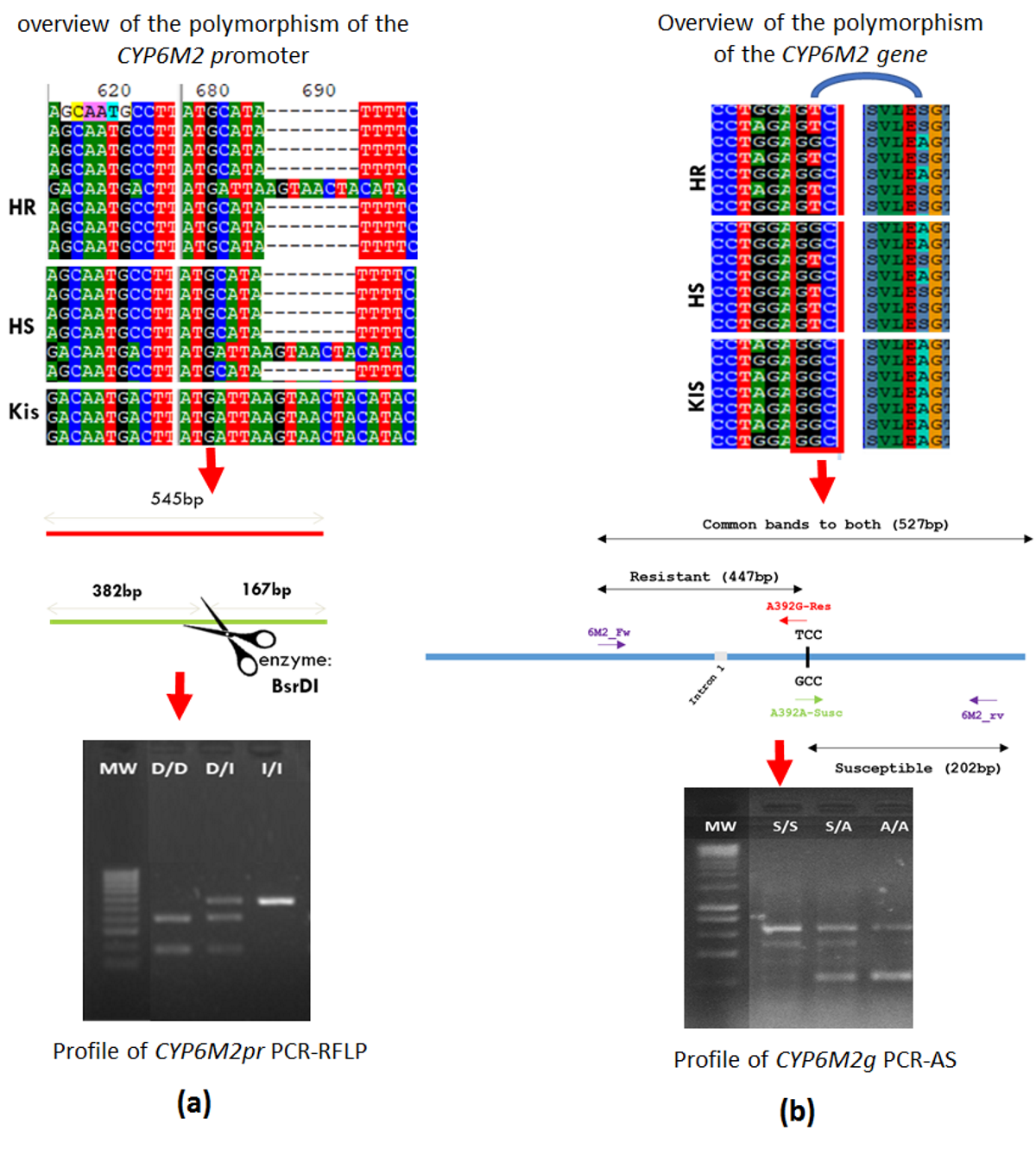

Supplement: Supplementary file 1 [file genes-14-00052-s001.zip › Figure S11. Representative diagram of DNA-based assay to genotype a keys mutation in An. gambiae CYP6M2.tif]

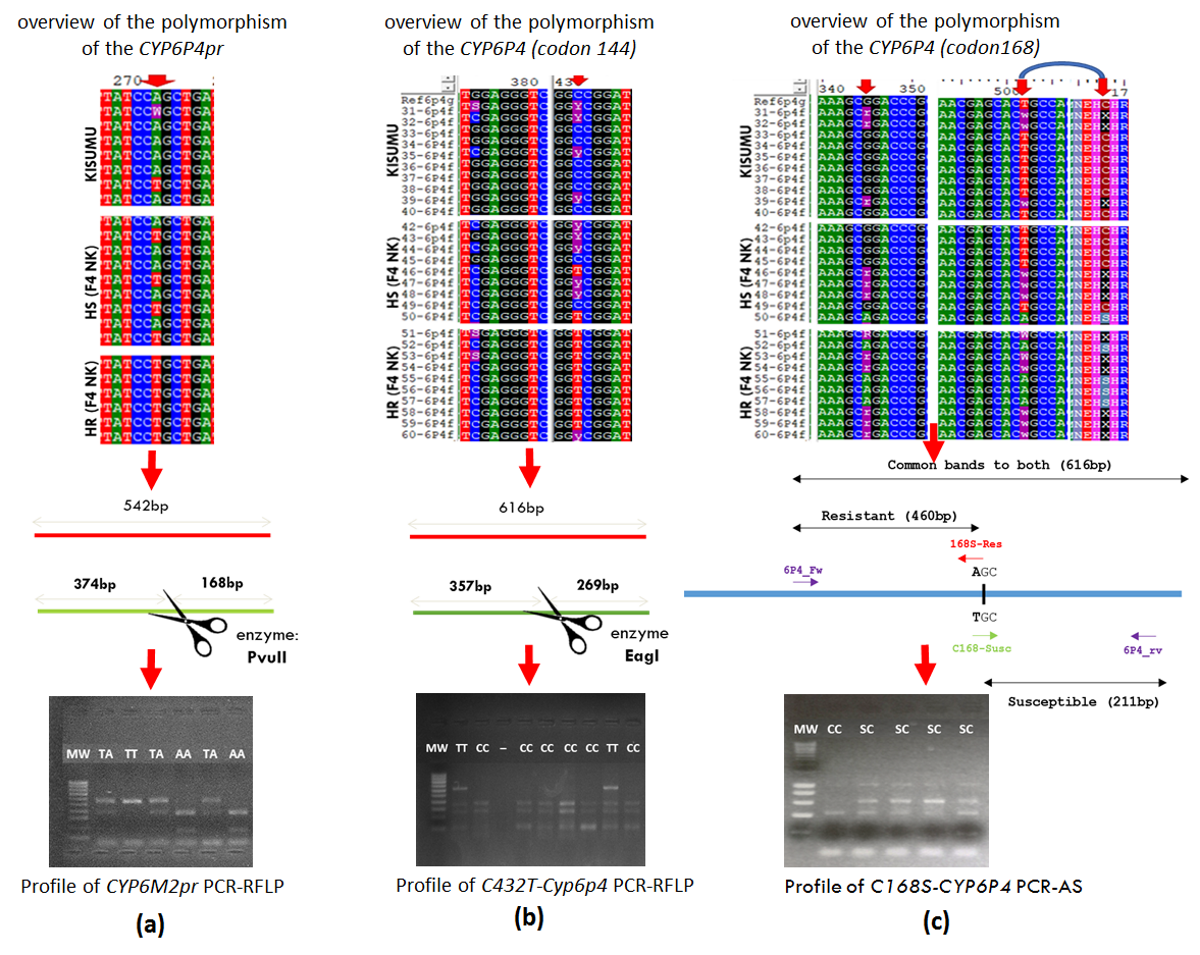

Supplement: Supplementary file 1 [file genes-14-00052-s001.zip › Figure S12. Representative diagram of DNA-based assay to genotype a keys mutation in An. gambiae CYP6P4.tif]

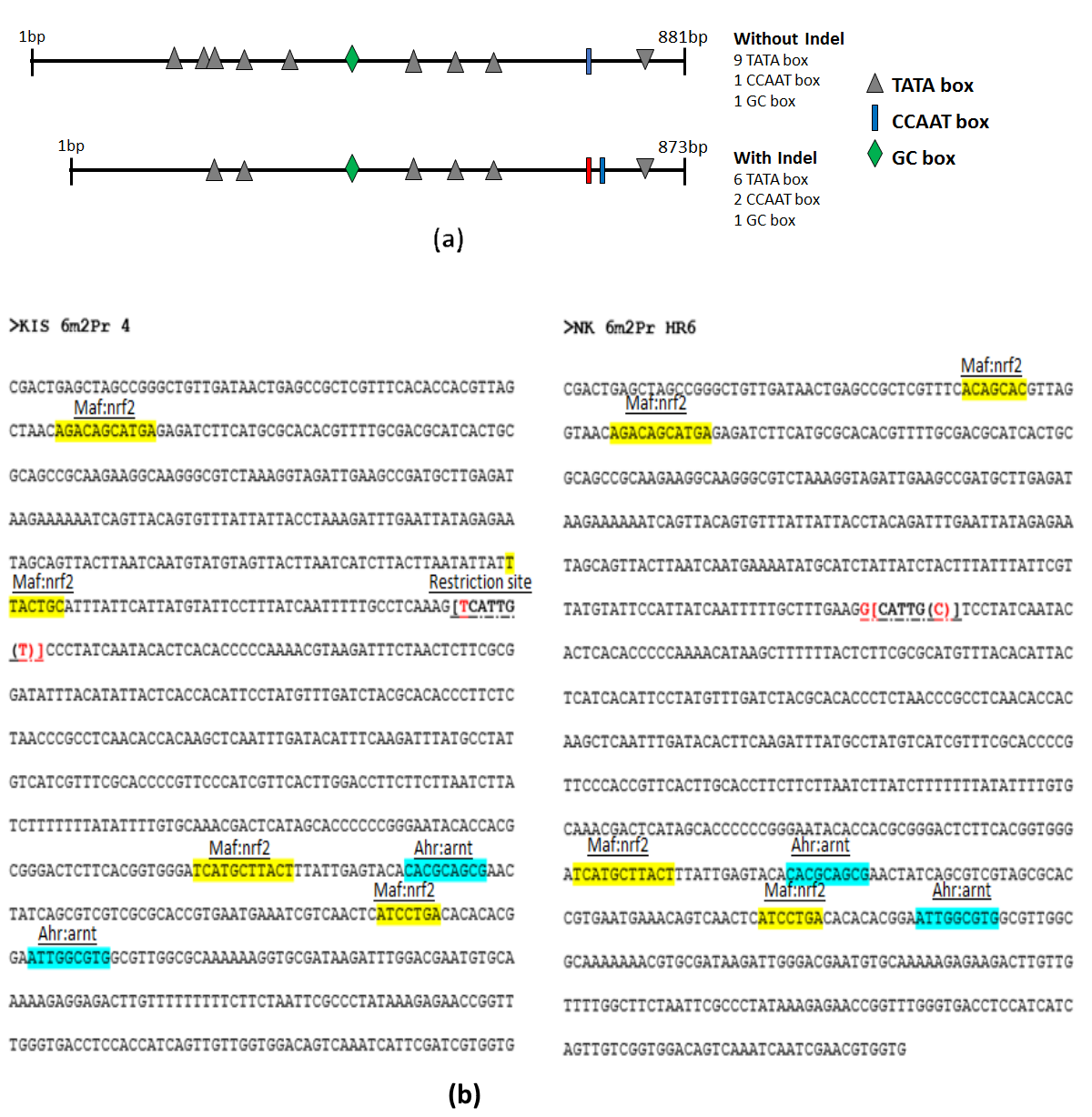

Supplement: Supplementary file 1 [file genes-14-00052-s001.zip › Figure S1. Nucleotide sequence of the 881bp of upstream region of CYP6M2 showing (a) the regulatory sequences identify by GPMiner and (b) the transcription factors binding sites using Alggen.tif]

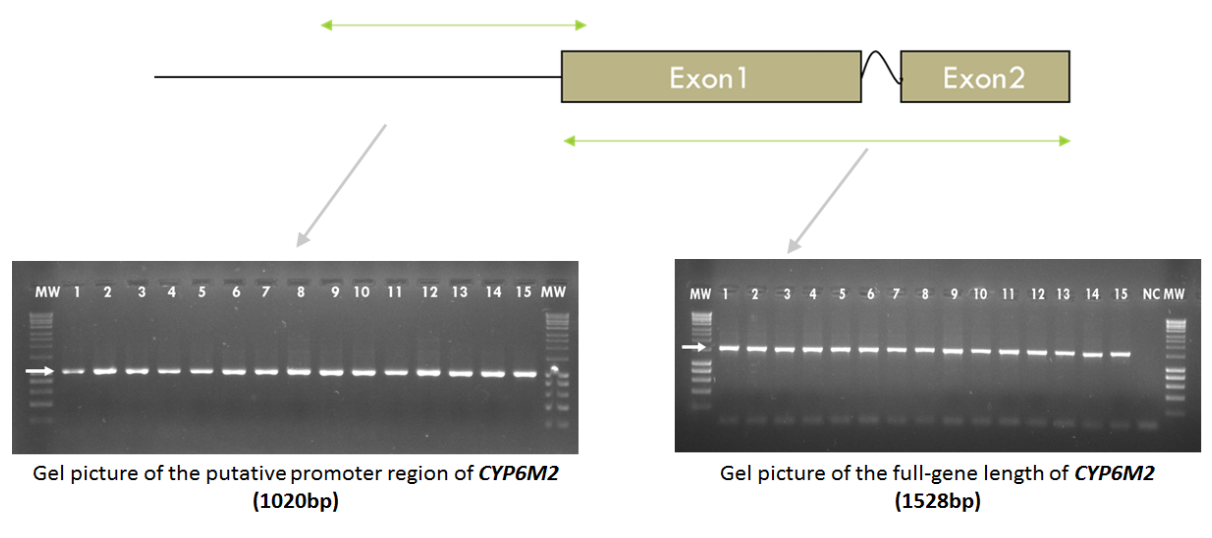

Supplement: Supplementary file 1 [file genes-14-00052-s001.zip › Figure S2. PCR amplification of the upstream and full-gene region of CYP6M2.tif]

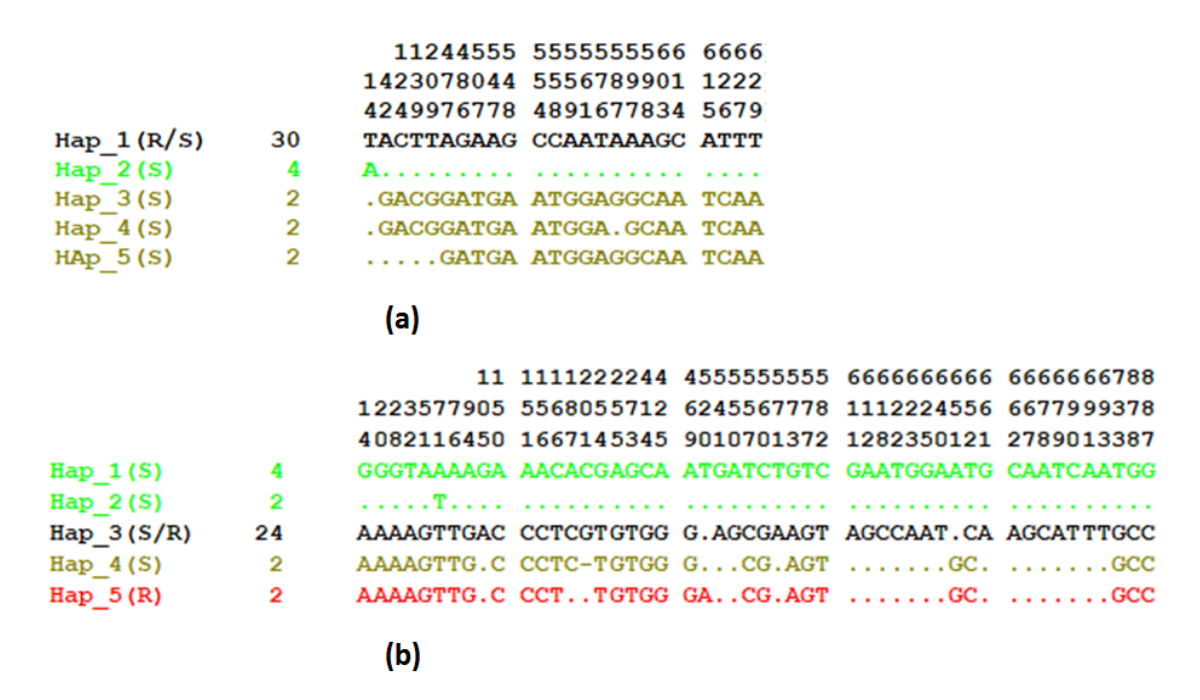

Supplement: Supplementary file 1 [file genes-14-00052-s001.zip › Figure S3. Polymorphic sites and haplotypes of the CYP6M2 upstream region in (a) An. coluzzii and (b) An. gambiae hybrid from F4.tif]

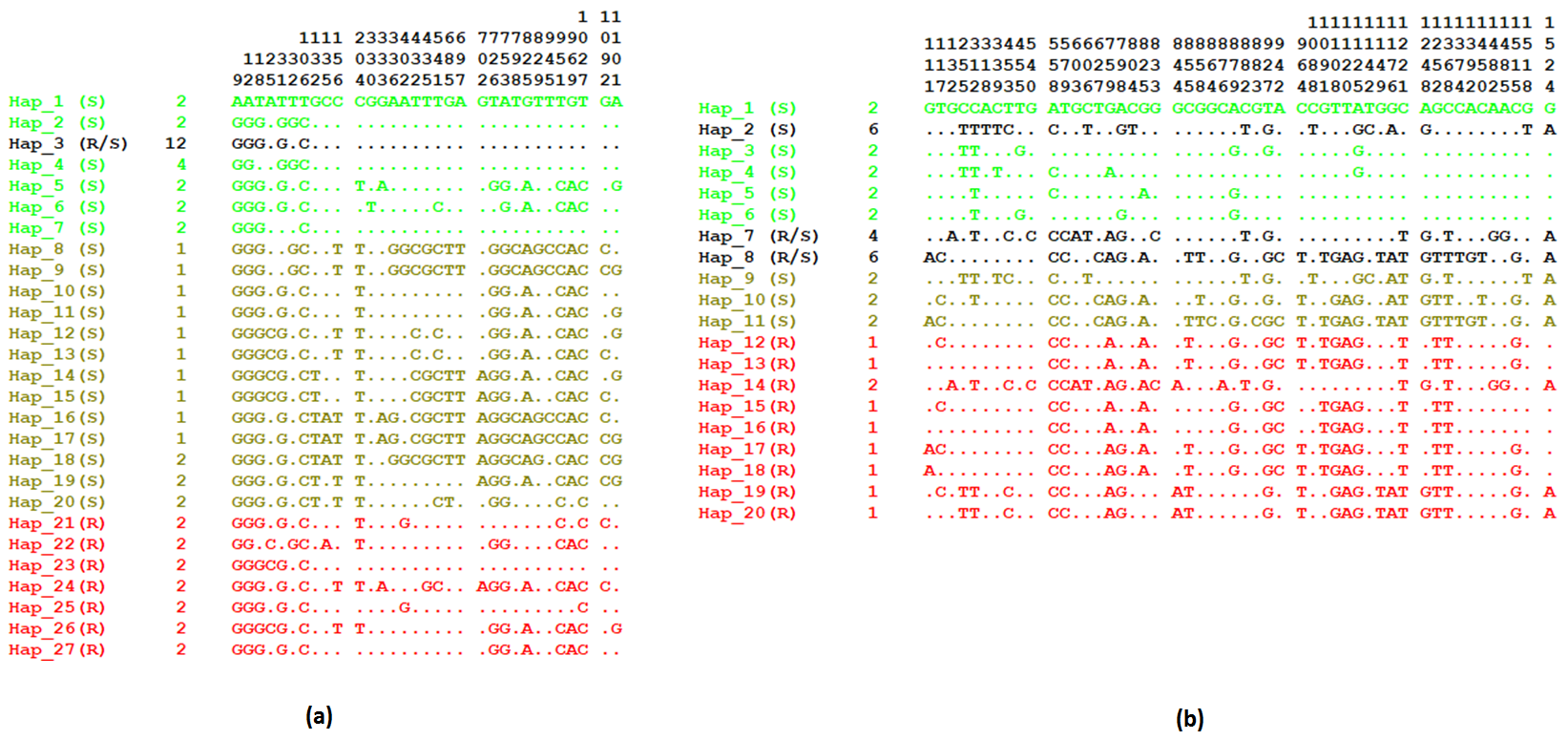

Supplement: Supplementary file 1 [file genes-14-00052-s001.zip › Figure S4. Polymorphic sites and haplotypes of the full –gene length of CYP6M2 in (a) An. coluzzii and (b) An. gambiae hybrid from F4.tif]

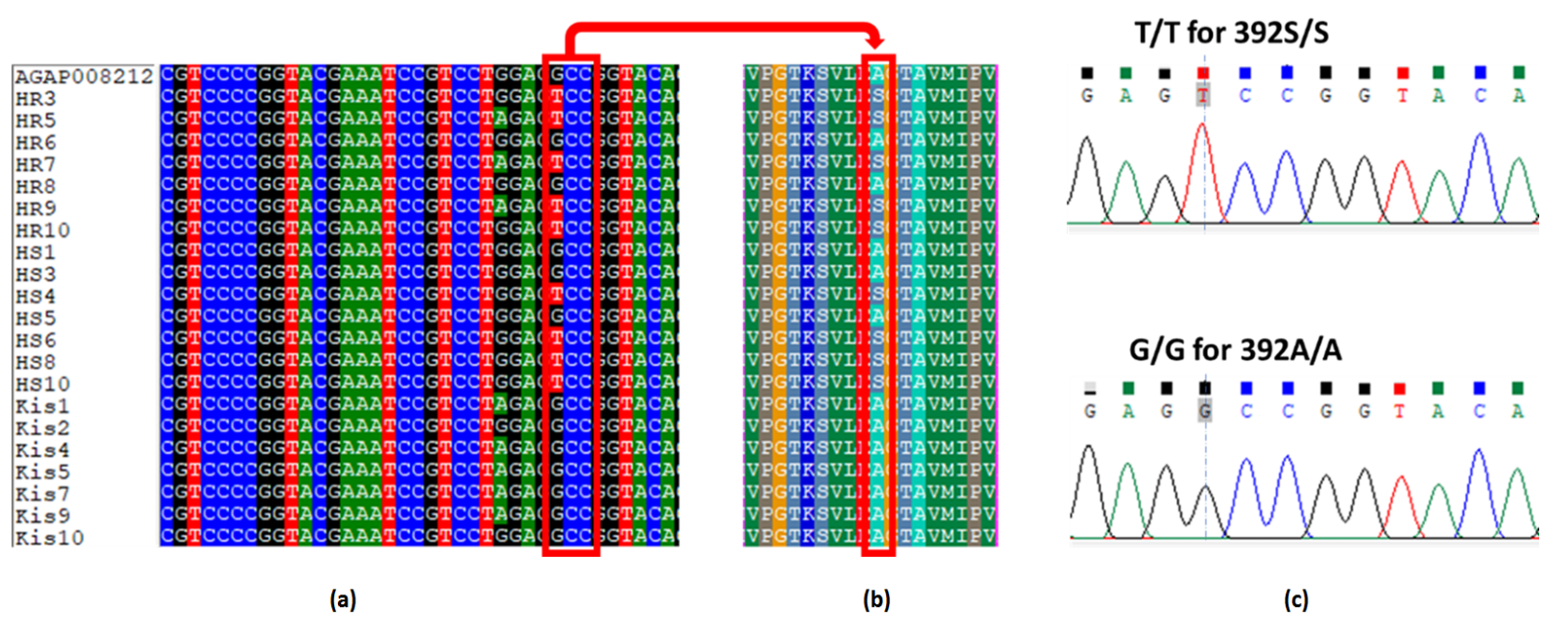

Supplement: Supplementary file 1 [file genes-14-00052-s001.zip › Figure S5. Sequencing of the portion of the full CYP6M2-gene length spanning the A392S mutation.tif]

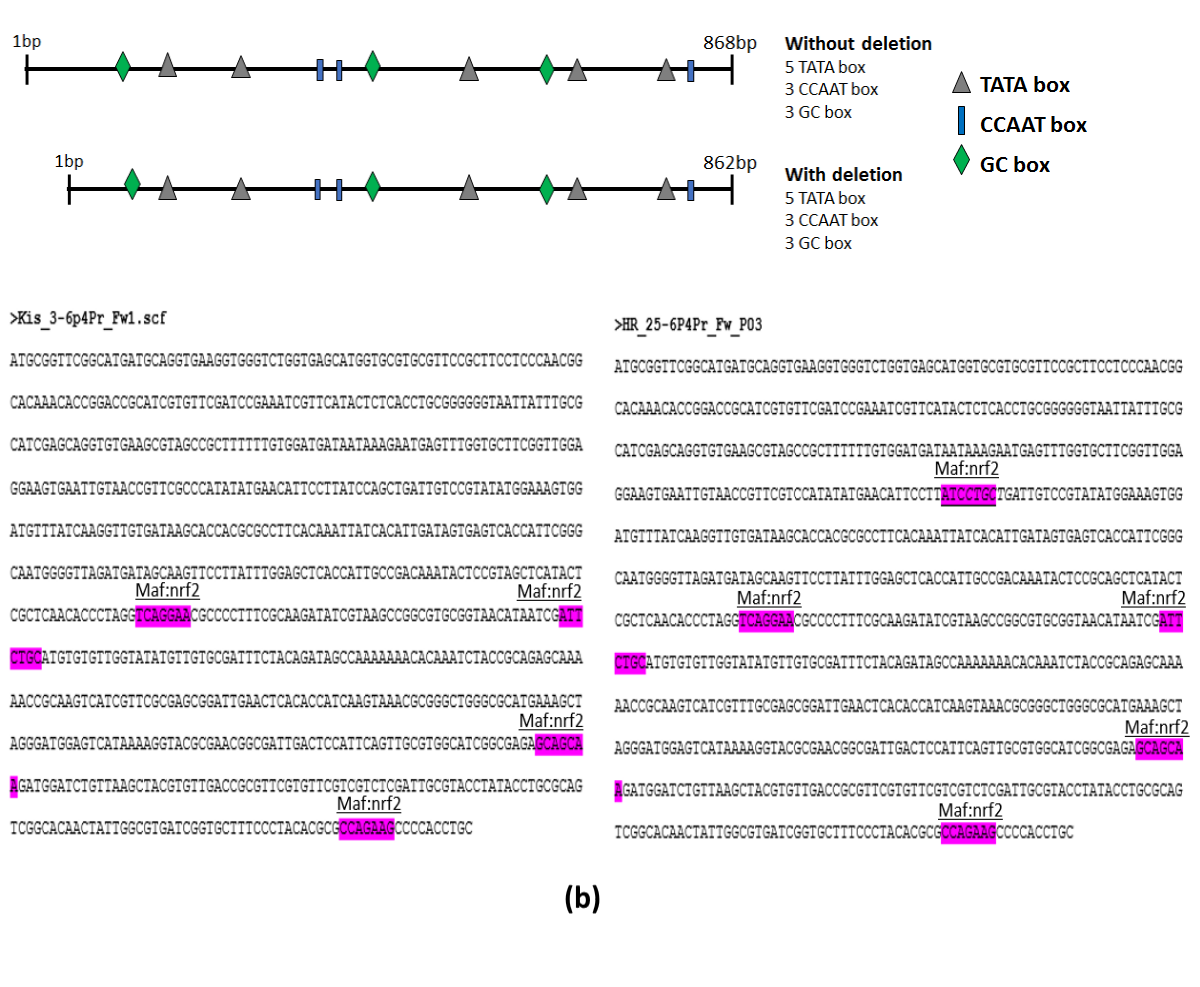

Supplement: Supplementary file 1 [file genes-14-00052-s001.zip › Figure S6. Nucleotide sequence of the 868 bp of the upstream region of CYP6P4 showing (a) the regulatory sequences identify by GPMiner and (b) the transcription factors binding sites using Alggen.tif]

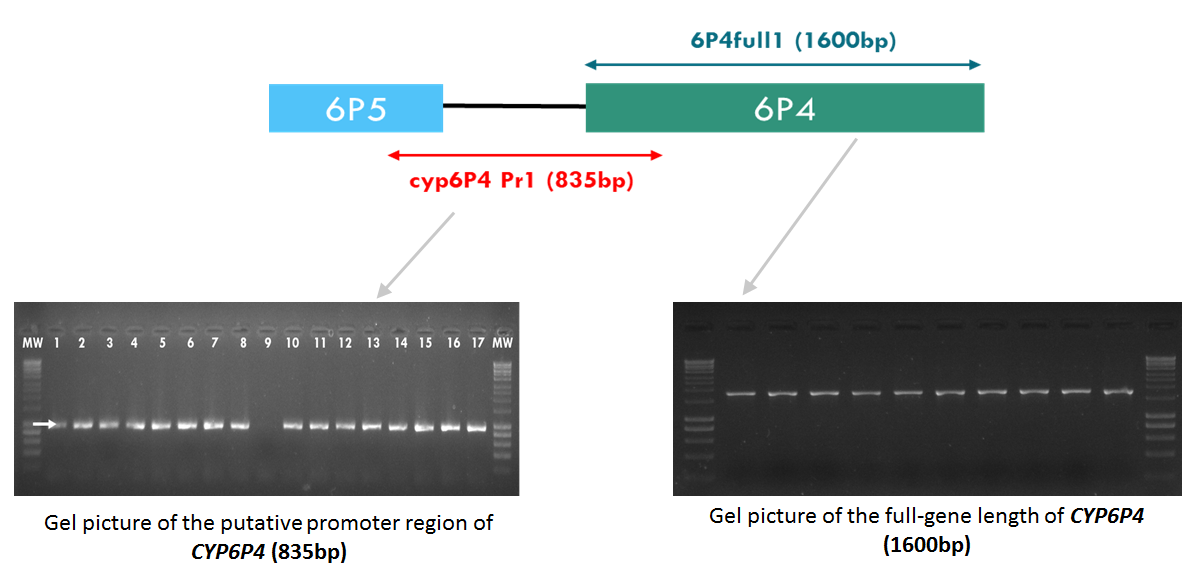

Supplement: Supplementary file 1 [file genes-14-00052-s001.zip › Figure S7. PCR amplification of the upstream and full-gene region of CYP6P4.tif]

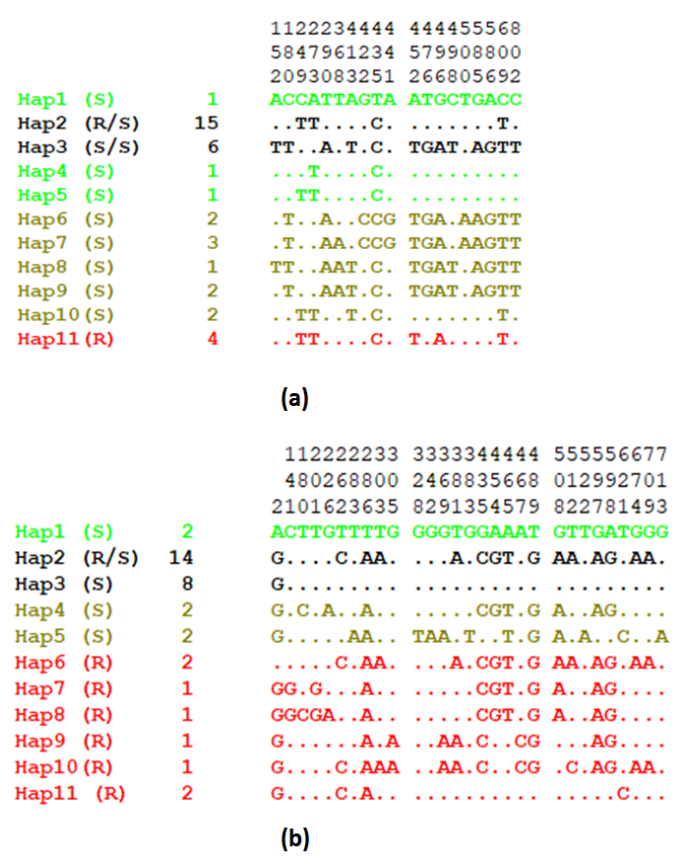

Supplement: Supplementary file 1 [file genes-14-00052-s001.zip › Figure S8. Polymorphic sites and haplotypes of the CYP6P4 upstream region in (a) An. coluzzii and (b) An. gambiae hybrid from F4.tif]

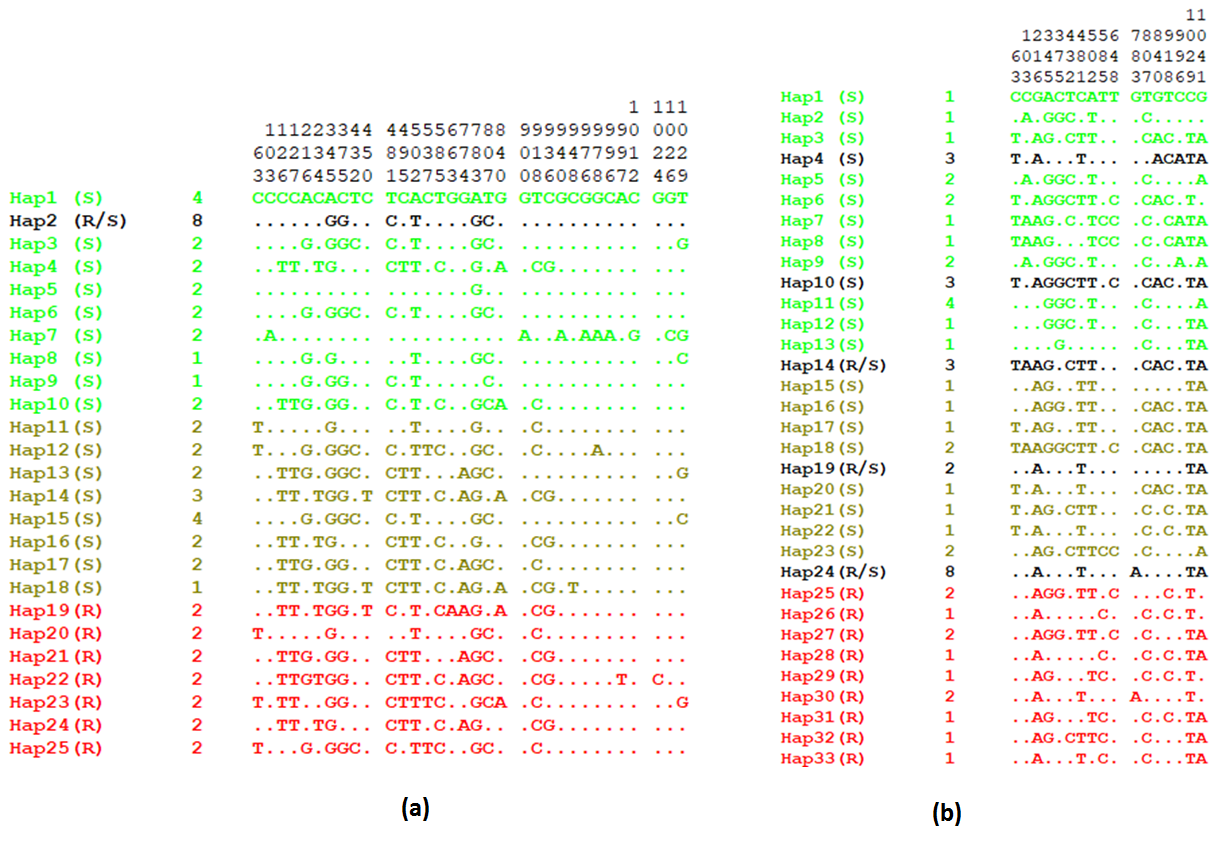

Supplement: Supplementary file 1 [file genes-14-00052-s001.zip › Figure S9. Polymorphic sites and haplotypes of the 1,051bp fragment of CYP6P4 gene in (a) An. coluzzii and (b) An. gambiae hybrid from F4.tif]

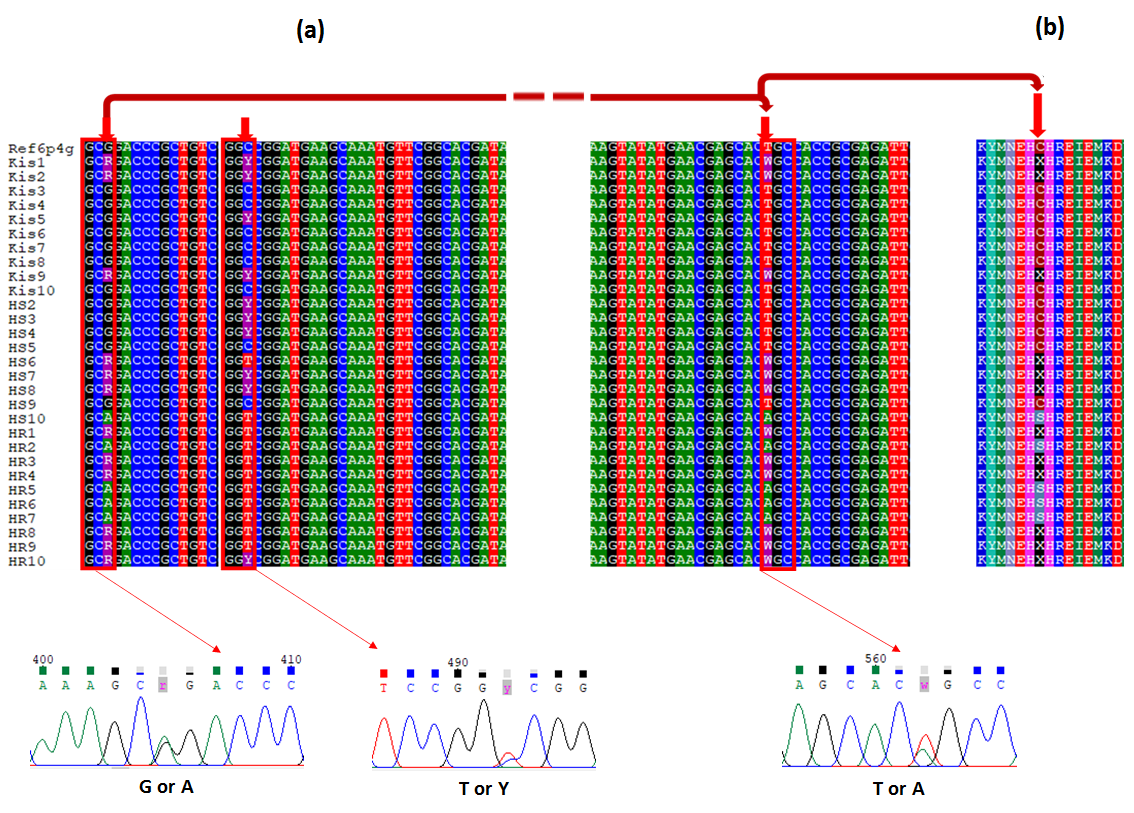

Supplement: Supplementary file 1 [file genes-14-00052-s001.zip › Figure S10. Sequencing of the portion of the full CYP6P4-gene length spanning the all the mutation found.tif]
